# Whole body oxygen uptake and evoked knee torque in response to low frequency electrical stimulation of the quadriceps muscles: $\overset{•}{V}O_{2}$ frequency response to NMES

**DOI:** 10.1186/1743-0003-10-63

**Published:** 2013-06-28

**Authors:** Conor M Minogue, Brian M Caulfield, Madeleine M Lowery

**Affiliations:** 1 School of Electrical, Electronic and Communications Engineering, University College, Dublin, Ireland; 2 School of Physiotherapy & Performance Science, University College, Dublin, Ireland

**Keywords:** Electrical stimulation, Metabolic response, Muscle energetics

## Abstract

**Background:**

There is emerging evidence that isometric Neuromuscular Electrical Stimulation (NMES) may offer a way to elicit therapeutically significant increases in whole-body oxygen uptake in order to deliver aerobic exercise to patients unable to exercise volitionally, with consequent gains in cardiovascular health. The optimal stimulation frequency to elicit a significant and sustained pulmonary oxygen uptake has not been determined. The aim of this study was to examine the frequency response of the oxygen uptake and evoked torque due to NMES of the quadriceps muscles across a range of low frequencies spanning the twitch to tetanus transition.

**Methods:**

Ten healthy male subjects underwent bilateral NMES of the quadriceps muscles comprising eight 4 minute bouts of intermittent stimulation at selected frequencies in the range 1 to 12 Hz, interspersed with 4 minutes rest periods. Respiratory gases and knee extensor torque were simultaneously monitored throughout. Multiple linear regression was used to fit the resulting data to an energetic model which expressed the energy rate in terms of the pulse frequency, the torque time integral and a factor representing the accumulated force developed per unit time.

**Results:**

Additional oxygen uptake increased over the frequency range to a maximum of 564 (SD 114) ml min^-1^ at 12 Hz, and the respiratory exchange ratio was close to unity from 4 to 12 Hz. While the highest induced torque occurred at 12 Hz, the peak of the force development factor occurred at 6 Hz. The regression model accounted for 88% of the variability in the observed energetic response.

**Conclusions:**

Taking into account the requirement to avoid prolonged tetanic contractions and to minimize evoked torque, the results suggest that the ideal frequency for sustainable aerobic exercise is 4 to 5 Hz, which coincided in this study with the frequency above which significant twitch force summation occurred.

## Introduction

Neuromuscular Electrical Stimulation (NMES) is widely used to activate muscle in a rehabilitation setting for recovery and maintenance of muscle performance [1]. The objective in such applications is often to create repeated high intensity tetanic muscle contractions each lasting several seconds, however, typical stimulation regimes do not evoke a therapeutically significant increase in whole-body oxygen demand [2-5], and so are unsuitable as a means of delivering cardiovascular exercise to patients unable to move voluntarily. There are many situations in which such an option would be desirable; for example in individuals with exercise limitations due to spinal cord injury, joint disease or impaired cardio-pulmonary function.

Exercise intensities in the range 3 to 5.9 METs, accumulating to 10 MET h wk^-1^, are associated with reduced cardiovascular disease and reduced premature mortality [6]. Patients with reduced functional capacity may benefit from shorter bouts of less intense exercise, nonetheless, a viable NMES-induced aerobic exercise must be capable of creating a sustained energy demand at a rate which is a multiple of the resting rate [7]. Functional electrical stimulation (FES) assisted cycling or rowing has been shown to produce therapeutically significant increases in oxygen uptake in people with spinal cord injury [8], however this approach may not be suitable for neurologically intact patients due to a higher sensitivity to stimulation induced pain and/or reflex responses [9]. In recent years there have been several investigations into the efficacy of isometric NMES to increase the functional capacity of the cardio-pulmonary system, as well as the exercise capacity of the leg muscles, in patients with heart failure [10-12] and pulmonary disease [13,14]. There is, however, no apparent consensus on the optimum stimulation parameters which should be deployed. Dobsack *et al.* have shown that an 8 week NMES training intervention based on a frequency of 10 Hz increased the functional performance and aerobic capacity of chronic heart failure patients [10]. Nuhr *et al.* have similarly shown that an NMES regime at 15 Hz applied for 4 hours per day increased aerobic capacity in chronic heart failure patients [12]. Muscle biopsies indicated biochemical markers and structural changes in the muscle consistent with increased oxidative capacity. Vivodtzev *et al.* treated severely deconditioned COPD patients with an alternating pattern of 35 Hz and 5 Hz in a stimulation session lasting 30 minutes. After 4 weeks training there were significant improvements in dyspnea during everyday tasks, and in walking distance, compared to controls. [14] Bourjeily *et al.*, also working with COPD patients, showed an increase in muscle performance, but no increase in aerobic capacity, following a 6 week NMES training intervention using 50 Hz stimulation [13]. None of these studies reported the aerobic exercise intensity achieved with the stimulation pattern used.

Experimental measurements of steady state oxygen consumption during isometric NMES at tetanic stimulation frequencies have shown metabolic levels of approximately 2 MET [2-5,15], with rapid fatigue such that the exercise cannot be sustained. Theurel *et al.* measured whole body oxygen uptake during repeated high intensity quadriceps contractions at 46% MVC and found that the total $\dot{V}O_{2}$ was approximately twice resting levels, or less than 20% of $\dot{V}O_{2\mathrm{MAX}}$[2]. Similar levels of oxygen uptake were observed with intermittent tetanic isometric contractions of the large leg muscles at 20 Hz [15], while $\dot{V}O_{2}$ levels four times resting levels were reported during dynamic quadriceps NMES [16]. In general, results suggest that stimulation patterns with greater number of contraction cycles per minute tend to give rise to higher oxygen consumption rates [2-5,15-19]. This is consistent with the findings of Russ *et al*. who used 31P NMR spectroscopy to study muscle metabolism and found that it was energetically more costly to develop force in the muscle than to maintain it [20]. While the clinical results from using intermittent isometric tetanic patterns appear promising [10,12-14], the aerobic exercise intensities achieved with this form of stimulation may not reach preferred therapeutic levels. Consequently, optimization of the stimulation parameters to increase the oxygen uptake may yield further clinical benefits.

Recently, the use of high intensity isometric stimulation of the leg muscles at sub-tetanic frequencies (4 to 5 Hz), which results in intense force oscillation of the muscle, has been demonstrated to evoke substantial and prolonged whole-body oxygen uptake [18,21]. Exercise intensities in the range 50% to 60% of $\dot{V}O_{2\mathrm{MAX}}$, which can be sustained for up to an hour at acceptable levels of comfort, have been reported in a group of 16 healthy subjects [22]. This same technique has been used as a daily training intervention over several weeks and aerobic fitness improvements have been demonstrated in healthy subjects [23,24], patients with heart failure [17] and spinal cord injury [25].

The question remains as to what is the best stimulation frequency for eliciting sustained aerobic exercise in an isometric mode, given that it is also desirable to minimize the evoked muscle force so that the need for limb restraint can be avoided. The purpose of this study was to investigate muscle torque output and whole body oxygen consumption in response to NMES across a range of low stimulation frequencies, and thereby characterise the energetic cost of torque production as a function of frequency in this range. The primary hypothesis was that energy utilisization per unit time is related to the pulse frequency, the torque time integral and a factor representing the accumulated incremental torque in the period. To test this hypothesis the whole body energy rate, as measured by indirect calorimetry, was fitted to a multiple linear regression model based on a classical description of the different energetic components that arise during muscle contraction [26].

## Materials and methods

### Subjects

10 healthy male subjects took part in the study (Table 1). The study and the experimental protocols were approved by the Human Research Ethics committee of University College, Dublin. All subjects gave written informed consent.

**Table 1:** Summary demographic data for the group, N=10, male subjects

	**Mean**	**SD**	**Range**	**Units**
**Age**	35.0	11.1	22 - 53	y
**Bodymass**	78.8	14.4	58 - 104	kg
**BMI**	24.0	2.8	20.8 - 28.7	kg m^-2^
**MVC**	256.6	64.6	163 - 379	N m

### Experimental setup

All subjects fasted overnight and had a standard breakfast comprising 40 g cereal and 100 ml whole milk, approximately 1 hour before the test. Subjects were seated in a dynamometer (Cybex , USA) that was set-up for measurement of isometric knee extensor torque of the right leg at 60° knee flexion. The left leg was also restrained isometrically at approximately the same angle. The torque signal from the dynamometer was continuously recorded using a data acquisition system (Biopac, USA) at a sampling rate of 200 Hz. Two large, (12 cm × 16 cm), hydrogel stimulation electrodes, (Axelgaard, USA) were applied to the quadriceps of each leg and connected to a research stimulator, (NT2010, Biomedical Research Ltd., Ireland), which was programmed to produce in each leg a constant current, symmetric biphasic, square wave pulse train at up to 200 mA peak, with a phase duration of 600 *μ*s and an interphase interval of 100 *μ*s. While the pulses comprised two symmetric phases of opposite polarity, the polarity of the leading phase was reversed on each successive pulse to avoid any direct current bias. While both legs were stimulated at the same time, pulses to the right and left legs were time-multiplexed so as not to occur simultaneously. Energy usage was estimated by indirect calorimetry; the subject was fitted with a face mask connected to a pulmonary gas analysis system (Quark, Cosmed Italy) which allowed breath-by-breath estimation of the volume of oxygen consumed and carbon-dioxide produced. In addition to the breathing rate *R*_*f*_, the following gas exchange responses were collected: minute oxygen uptake, $\dot{V}O_{2}$ ventilation ${\dot{V}}_{E}$, respiratory exchange ratio (*RER),* and the ventilation equivalent for oxygen. ${\dot{V}}_{E}/\dot{V}O_{2}$ Prior to measurement of each subject, the gas analysis flow meter was calibrated with a standard 3 liter syringe and the gas concentration sensors were calibrated using a certified calibration gas.

Subjects began by performing 3 maximum voluntary contractions (MVC) of the right leg, each of approximately 5 seconds duration, separated by 60 to 90 seconds and the highest value was taken as their MVC. Next, the highest tolerated stimulus intensity was determined by setting the stimulator pulse frequency to 12 Hz, with an intermittent pattern of 5 seconds on and 5 seconds off, which corresponded to the most demanding pattern in the subsequent experiment. An intermittent stimulation pattern was used because previous pilot testing with continuous pulse trains indicated that some subjects could not tolerate continuous pulse trains at 6 Hz and above, even though they could readily tolerate 4 Hz indefinitely at the same stimulation intensity. The stimulus intensity was increased slowly until the subject indicated the highest level they could readily tolerate for 4 minutes. This level was noted and used for all the subsequent sequences.

Pilot testing indicated that subjects reached a steady state with regard to $\dot{V}O_{2}$ uptake within 4 minutes. The stimulation sequence therefore comprised 8 bouts of intermittent NMES, each bout lasting 4 minutes, with a rest interval of 4 minutes between each bout. The whole sequence therefore lasted 60 minutes. The pulse frequency in each of the 8 bouts was 1, 2, 4, 5, 6, 8, 10 and 12 Hz, respectively. The non-linear distribution of test frequencies over the range was chosen because pilot testing had indicated that partial twitch fusion began to occur in the range 4 to 6 Hz. The term ‘partial fusion’ is used here to indicate that the twitch torque had not returned to zero before the next twitch occurred.

The protocol started with a rest period of 4 to 5 minutes to establish the subject’s resting $\dot{V}O_{2}$. The stimulation intensity was set to the subject’s previously determined tolerable level and the sequence was begun. After completion of the 60 minute stimulation sequence, recording of respiratory gas parameters continued for approximately 3 minutes.

### Data analysis

For each subject, the mean amplitude of the peak torque (*T*), the torque-time-integral (*TTI*) and the mean $\dot{V}O_{2}$ and RER were estimated for the last 60 seconds of each 4 minute bout. The *TTI* was estimated as the sum of all samples in the period, multiplied by the sampling interval. *R*_*f*_ , ${\dot{V}}_{E}$ and the ventilation equivalent for oxygen ${\dot{V}}_{E}/\dot{V}O_{2}$ were averaged over the last 10 breaths of each period. In addition, a measure representing the amount of force development was devised as follows: the torque waveform was differentiated with respect to time to give a variable representing the rate of change of torque, *dT/dt*. The negative component of this function was removed, leaving only that part representing a positive rate of torque development, and this was then integrated over the last 60 seconds of each stimulation bout:

$$\mathrm{dTTI}=\int_{180}^{240}\;\max\left(\frac{\mathrm{dT}}{\mathrm{dt}},0\right)\mathrm{dt}$$

For each subject, the resting $\dot{V}O_{2}$ rate was subtracted from each of the estimates of $\dot{V}O_{2}$ during the test bouts to give the net increase due to the electrical stimulation. The respiratory exchange ratio (*RER*) was estimated as the quotient of molar volumes CO_2_/O_2,_ on a breath by breath basis. The signal to noise ratio of this signal was poor at low $\dot{V}O_{2}$ rates. An overlapping moving average of 9 successive breaths was used to smooth each signal before evaluation of the respiratory exchange ratio. The estimates of energy rate were provided by the respiratory gas analysis system through indirect calorimetry.

### Model

In the context of a standard Hill-type model, the overall energy rate in a muscle contraction is typically described in the following terms [26,27].

(1) $$\dot{E}={\dot{h}}_{a}+{\dot{h}}_{m}+{\dot{h}}_{s}+{\dot{h}}_{w}$$

$\dot{E}$ is the total heat rate, expressed in W kg^-1^$\;{\dot{h}}_{a}$ is the activation heat, and represents the energy associated with the release and uptake of calcium from the sarcoplasmic reticulum and its binding and release from troponin. It is modeled as a nonlinear function of the pulse frequency [27], however, it is essentially linear over the restricted frequency interval considered here (1 to 12 Hz) ${\dot{h}}_{m}$ is the maintenance heat rate and represents the energy associated with cross-bridge cycling in the maintenance of force. For isometric contractions it can be modeled as linear function of the force [27]. ${\dot{h}}_{s}$ is the shortening heat rate which is normally expressed as being proportional to the absolute shortening velocity [28]. In the isometric situation the external shortening velocity is zero, albeit that the contractile element (CE) of the Hill model shortens in developing force through the series elastic element (SEE). Hill proposed that the heat of shortening was proportional to the distance shortened, independent of the force:

(2) $$\mathrm{\Delta H}=a\left(-\mathrm{\Delta L}\right)$$

where, ∆*H* is amount of heat liberated and ∆*L* is the distance shortened. The coefficient *a* is itself a function of muscle length and the muscle active state [26]. Assuming a linear force-length relationship for the SEE, then Equation 2 leads simply to:

(3) $$\mathrm{\Delta H}=a\left(\mathrm{\Delta F}\right)$$

where *ΔF* is the increment in force. This suggests that in isometric mode the heat of shortening can be represented as a heat of force development, so that each positive excursion of the force has an energy cost. ${\dot{h}}_{w\;}$ is the external mechanical work rate produced by the muscle. Since this is zero for the isometric situation, the overall energy rate in this case can be written in the following form:

(4) $$\dot{E}=c_{1}f+c_{2}F+c_{3}{\dot{F}}_{+}$$

where, *c*_*1*_*, c*_*2,*_*c*_*3*_ are coefficients to be identified and *f* is the pulse frequency. ${\dot{F}}_{+}$ represents the rate of change of force with respect to time while the force is increasing and is defined to be zero for decreasing force. The energy in a unit time interval is the time-summated version of this equation, leading to an equation of the following form

(5) $$\dot{E}=c_{1}f+c_{2}\mathrm{FTI}+c_{3}\mathrm{dFTI}$$

*FTI*, the force time integral, represents the integral of the force generated over the time period in question and *dFTI* represents the accumulated positive force excursions in the time period. This study measured knee extensor torque, rather than muscle force, therefore the corresponding expression is

(6) $$\dot{E}=c_{1}f+c_{2}\mathrm{TTI}+c_{3}\mathrm{dTTI}$$

where, *TTI* is the torque time integral and *dTTI* is the accumulated incremental torque.

### Statistical analysis

Descriptive statistics (mean and standard error) of the gas exchange variables as well as *R*_*f*_, *T*, *TTI , dTTI* and energy Rate $\dot{E}$ , were calculated for each frequency of stimulation. The coefficients *c*_*1*_*, c*_*2*_ and *c*_*3*_ of Equation 6 were estimated by multiple linear regression, whereby the energy rate was selected as the observable and the pulse frequency, *f, TTI* and *dTTI* as predictors. Post hoc ANOVA calculations were carried out to test for an effect of frequency on $\dot{E}$ and *RER* in subdivisions of the frequency range. Statistical calculations were carried out using Minitab 15 (Minitab Inc., USA).

## Results

**Figure 1:**
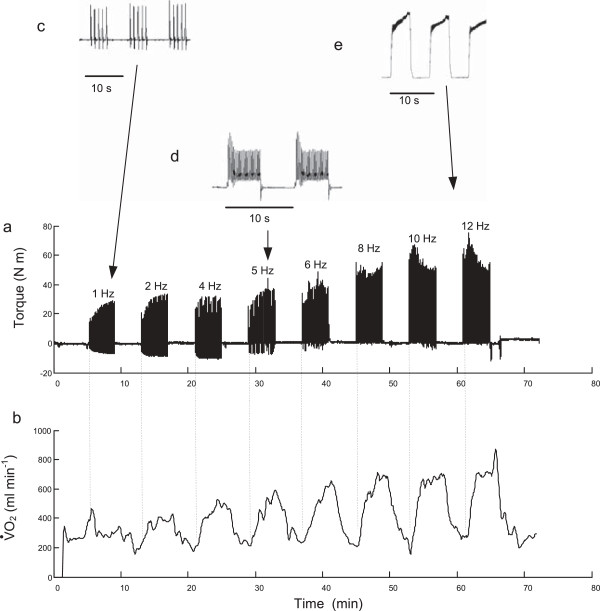
**Sample torque and oxygen uptake record. (a)** Measured joint torque and **(b)** rate of oxygen consumption $\dot{V}O_{2}\;$ for a representative subject over the 60 minute test period showing 4 minute bouts of NMES at selected frequencies. Inserts, **(c through e)**, show torque responses on an enlarged time-scale so that partial twitch fusion can be seen.

**Figure 2:**
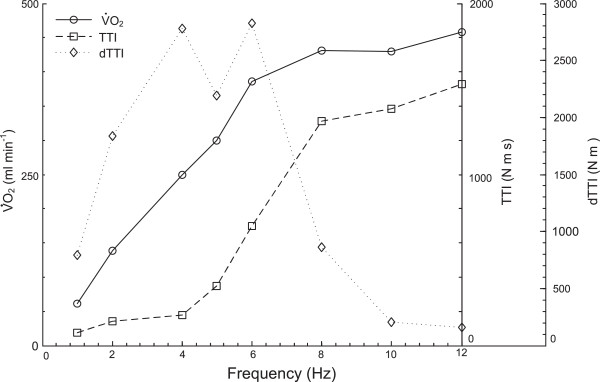
**Sample time-averaged oxygen uptake and torque functions.** Average $\dot{V}O_{2}$_,_*TTI*, and *dTTI* for the last 60 seconds of each bout, as a function of stimulation frequency, for a typical subject.

Figure 1 presents the measured joint torque and oxygen consumption responses for a typical subject during a session. The increasing degree of force fusion as the impulse frequency increases is evident, Figure 1a-e. During the first stimulation bout at 1 Hz, individual force twitches can be seen. At a pulse frequency of 5 Hz, in this subject, there is a small degree of force fusion while at 12 Hz there is almost complete fusion with very little force “ripple” during the 5 second contraction time. The amplitude of the torque signal is approximately the same for the lower frequency bouts (1 through 4 Hz) but increases as force summation between twitches develops. The maximum twitch amplitude for this subject at 1 Hz is approximately 28 N-m, which represents 14% of his maximum voluntary torque. There is also evidence of the “staircase phenomenon” [29], in which the amplitude of the torque twitch increases (or decreases) with successive low frequency twitches. Figure 1b shows that the oxygen consumption increases and tends to stabilise towards the end of each four minute stimulation bout, while returning towards resting levels between each bout. The peak oxygen level attained increases with frequency, but the rate of increase declines towards the upper end of the frequency range. The torque functions *TTI* and *dTTI*, for the same subject as shown in Figure 1, are presented in Figure 2.

**Figure 3:**
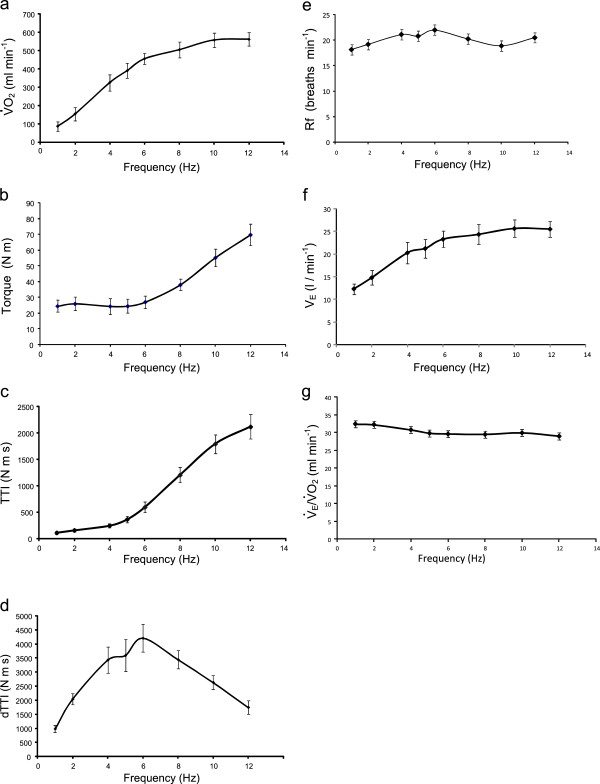
**Group mean pulmonary responses and torque functions.** Group mean (SEM) for the following responses as a function of stimulation frequency: **(a)** Additional $\dot{V}O_{2}$ over resting levels for the last 60 seconds of each bout, **(b)** peak torque *T*, **(c)** integral of torque, *TTI*, **(d)** integral of torque development, *dTTI*, **(e)** breathing frequency, *Rf ,***(f)** ventilation, ${\dot{V}}_{E}$**(g)** ventilation equivalent for oxygen, $${\dot{V}}_{E}/\dot{V}O_{2}.$$

The corresponding data for the group is shown in Figure 3, where Figure 3a shows the group mean additional $\dot{V}O_{2}$ above resting levels at each frequency, and Figure 3b through d show the group mean torque functions *T* , *TTI* and *dTTI*, respectively. Figure 3e through g show the group mean breathing frequency *R*_*f*_, the ventilation ${\dot{V}}_{E}$ and ventilation equivalent for oxygen ${\dot{V}}_{E}/\dot{V}O_{2}$ at the end of each stimulation bout. The signal to noise ratio of ${\dot{V}}_{E}/\dot{V}O_{2}$ was poor at low levels of $\dot{V}O_{2}$ making a reliable estimate difficult at the very lower end of the frequency range, however the mean value at the end of each of the rest periods was 36.8 (SE 0.6). The group mean (SD) amplitude of stimulation was 87.6 (7.2) mA and the corresponding group mean of the root mean square (rms) current ranged from 3.1 mA at 1 Hz to 10.5 mA at 12 Hz.

**Figure 4:**
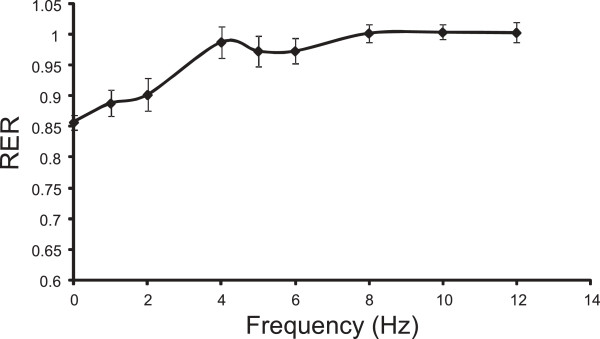
**Respiratory exchange ratio.** Group mean (SEM) for the Respiratory Exchange Ratio (RER), averaged over the final 60 seconds of each bout, as a function of stimulation frequency.

The group mean respiratory exchange ratio, RER, is plotted for each frequency in Figure 4. The mean resting value was approximately 0.85, and at stimulation frequencies of 4 Hz and above, the mean value appears to be approximately constant and close to unity. A post-hoc ANOVA shows no effect of frequency on the RER value above 4 Hz (p=0.61). After the termination of each NMES bout the RER value exceeded unity briefly before returning to a mean value of 0.97 (SE 0.02) by the end of the subsequent rest period, (averaged over the last 60 seconds of the 4 minute rest period).

The result of the multiple regression analysis is summarized in Equation 7, and the associated statistical data is in Table 2. All the coefficients of the linear model were estimated with a statistical significance of p < 0.05. The R^2^_adj_ value indicates that 88.3% of the variability in the data is accounted for by the regression equation.

(7) $$\dot{E}=-0.157+0.098f+0.000564\;\mathrm{TTI}+0.000334\mathrm{dTTI}$$

**Table 2:** **Regression analysis: energy as a function of *f TTI, dTTI***

**Predictor**	**Coefficient**	**SE**	**P value**
**Constant**	-0.16	0.11	0.147
***f***	0.10	0.02	<0.001
***TTI***	0.56 10^-3^	0.09 10 ^-3^	<0.001
***dTTI***	0.33 10^-3^	0.03 10 ^-3^	<0.001
**S = 0.354**	R^2^ = 88.7%	R^2^_adj_ = 88.3%	

**Figure 5:**
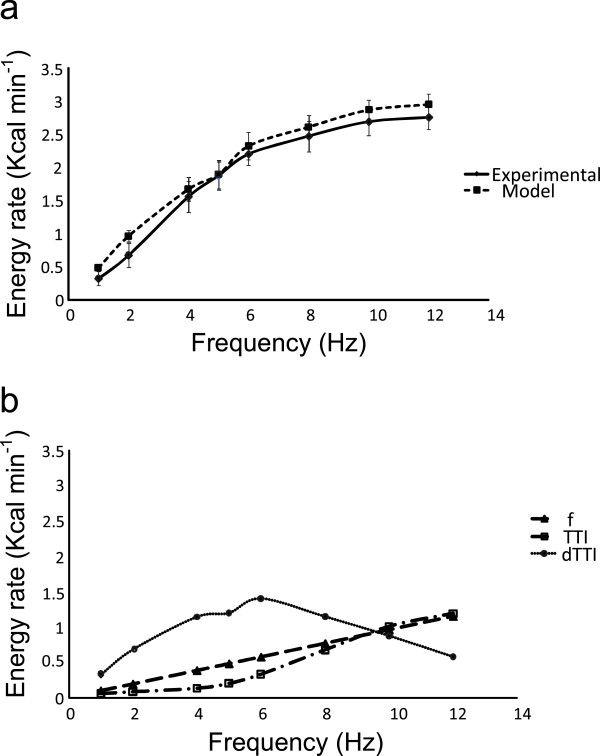
**Energetic model performance (a).** Comparison between the experimental group mean (SEM) energy rate and the group mean (SEM) energy rate predicted by the regression model of Equation 7. **(b)**. The contributions of the *f, TTI* and *dTTI* terms of the model.

A comparison between the experimental energy rate and the mean energy rate predicted by the model is summarized in Figure 5a. The energy rate appears to plateau at the upper end of the frequency range. This was confirmed by a post-hoc ANOVA with frequency as a factor over the three levels 8, 10 and 12 Hz which shows no significant effect of frequency (p=0.53). Figure 5b also shows the contribution of the *f, TTI* and *dTTI* terms of Equation 7 to the total energy rate.

## Discussion

This is the first study to simultaneously measure the torque output, whole-body oxygen uptake and energy utilisation during bouts of isometric electrical stimulation of the quadriceps muscle group at a selection of low pulse frequencies. Previous studies using various forms of intermittent isometric tetanic stimulation have reported low levels of additional oxygen uptake, [5,15] even at relatively high levels of force production [2]. This study found that although the additional oxygen uptake with unfused twitches was less than that at higher frequencies exhibiting higher degrees of twitch fusion, (see Figure 3a, 388 ml min^-1^ at 5 Hz compared to 564 ml min^-1^ at 12 Hz)), the unfused twitches can be tolerated continuously thus permitting a higher overall oxygen rate than tetanic stimulation which must be delivered intermittently. Morover, the unfused twitches result in lower peak forces than occur with tetanic stimulation.

There is an underlying assumption that the pulmonary oxygen uptake observed here was taken up by the stimulated muscles and not in other physiological compartments. The breathing rate did not increase excessively, Figure 3e, which otherwise might have suggested a sympathetic response to discomfort or anxiety, and there was no muscle work evident in other parts of the body, for example due, to bracing or altered posture. The oxygen uptake due to the respiratory muscles is also insignificant in the context of the additional whole body oxygen uptake [2].

This study did not specifically attempt to characterise the oxygen kinetics at the onset of each stimulation bout but inspection of the $\dot{V}O_{2}$ waveforms, such as Figure 1, suggest that the mono-exponential curve normally associated with volitional exercise may not be present. In particular, the rise-time at low frequencies would appear to be longer than would be expected for low intensity volitional exercise [30]. At higher frequencies the oxygen uptake increases rapidly at first, but instead of reaching a plateau continues to increase slowly. Kim *et al.* compared NMES induced leg extension with volitional exercise at the same 30W work level [16]. Unlike the plateau in $\dot{V}O_{2}$ observed with the volitional exercise after 10 minutes, the NMES induced $\dot{V}O_{2}$ continued to increase during the 1 hour session. These effects are likely to be due to differences in the way NMES activates the neuromuscular system compared to volitional exercise. Evidence suggests that NMES does not recruit motor units according to the physiological size principal and instead results in a more random selection, dependent on geometrical factors, axon diameter and orientation, which may have a higher proportion of type II fibres [16,31-33], some having a lower oxidative capacity.

It is also known that NMES leads to an exaggerated metabolic response with a bias towards anaerobic energy conversion, compared to equivalent voluntary exercise, with much greater depletion of PCr levels and increased acidity [34,35]. Prolonged tetanic contractions may restrict blood perfusion to the contracted fibers, effectively rendering them ischemic and preventing oxidative metabolism [36,37]. Relaxation phases between contractions are therefore required to restore blood supply. The oxygen uptake here at 5 Hz, though lower than the plateau level at 10 to 12 Hz, is still about 2.4 times resting levels. The relatively high oxygen uptake at this frequency is thus likely to be due to the combination of the energy expenditure in continuous shortening and lengthening of the contractile element and the absence of tetanic contraction compromising blood flow. Moreover, the sub-tetanic form can be tolerated continuously thus avoiding the need for relaxation phases which reduce the overall duty cycle.

The torque levels and the torque versus frequency characteristics observed here agree with those reported elsewhere. The mean (SD) twitch torque amplitude at 1 Hz was 24.4 (12.1) N m, see Figure 3b, which corresponds to mean transducer forces of 70 N. The torque amplitude varied little across the range 1 to 6 Hz. Binder Macleod *et al*. [38] reported similar twitch amplitudes of approximately 60 N when stimulating the quadriceps muscles. The torque ratio between 12 Hz and 1 Hz observed in the present study was 2.9:1, whereas a ratio of approximately 2.4:1 between 10 Hz and 1 Hz was reported by Binder Macleod *et al*[38].

The volumes of whole body oxygen consumed in the present study are relatively small in comparison to voluntary exercise, corresponding on average to about 3 MET at 12 Hz, see Figure 3a. In this study, stimulation was restricted to the quadriceps muscle group on each leg, to enable knee extensor torque to be used as an output measure. On the assumption that all the additional oxygen was taken up only in these muscles, then the oxygen uptake per kilogram of muscle is substantial. If the mean quadriceps muscle mass is assumed to be 2.5 kg per leg [16], then the additional oxygen uptake here, 561 ml min^-1^ at 12 Hz, would correspond to 112 ml min^-1^ kg^-1^ of muscle activated. This compares with a reported maximum oxygen uptake in muscle of 300 to 400 ml min^-1^ kg^-1^[16]. For a system designed to deliver therapeutic exercise, additional oxygen uptake could be stimulated by simultaneously activating the hamstring and gluteal muscles [21]. The stimulation intensity used in this study was limited by a maximal user tolerance of the 12 Hz stimulation and since users can tolerate higher phase charge levels at 4 or 5 Hz the energy rate would be higher than shown here for those frequencies. Also, the protocol used intermittent stimulation with a 50% duty cycle, because it was found that subjects could not tolerate continuous high intensity pulse trains above 6 Hz. The reduced tolerance to stimulation greater than 6 Hz may be associated with partial twitch fusion which begins to occur around that frequency. By involving more muscle mass through stimulation of more muscles, and limiting the frequency to 5 Hz so that a higher intensity and a higher duty cycle are tolerated, the whole body oxygen uptake can be considerably higher, as reported in previous studies [18,21,22].

The measured value of the respiratory exchange ratio *RER* is close to unity at frequencies of 4 Hz and above, Figure 4, suggesting a carbohydrate substrate. An alternative explanation for the elevated RER value could be that expired CO_2_ increased due to hyperventilation, however, there was no evidence of this in the ventilatory exchange for oxygen, see Figure 3e. This *RER* value is consistent with other observations in NMES induced exercise [2,15,16], which report higher carbohydrate usage than would occur during voluntary exercise at an equivalent exercise intensity. Kim *et al.* showed much greater depletion of muscle glycogen following quadriceps NMES, compared to volitional exercise of the same intensity. Both Hamada *et al.*[15] and Theurel *et al.*[2] have also reported RER levels close to unity at the start of an NMES session. At frequencies less than 4 Hz, the *RER* value is more representative of a lipid-carbohydrate mix, albeit that the oxygen uptake is quite low.

As hypothesised here, the steady state energy utilisation could be accounted for by an established model of muscle energy expenditure suitably adapted for isometric activity by replacing the heat of shortening by a factor representing the heat of incremental force development. The resulting expression, Equation 6, suggests that the twitch fusion characteristic of the muscle may be an important factor in determining the energy, and thereby oxygen, utilisation. At low pulse frequencies, where little or no force fusion takes place, and where the amplitude and force-time-integral of individual twitches are independent of frequency, Equation 6 suggests that the energy rate may be represented simply as a linear function of frequency, see Figure 3a. This is consistent with the finding that the ATP cost per twitch is independent of stimulation frequency in the sub-tetanic range [39], so that the ATP utilisation per unit time is a linear function of the pulse frequency.

Once partial twitch fusion occurs, then the force time integral and force development factors, *TTI* and *dTTI*, are no longer simple linear functions of frequency; see Figure 3c and (d). Although the maximum oxygen uptake occurs at the higher end of the frequency range the output torque is also higher due to the nonlinear temporal summation between twitches, Figure 3a. For a therapeutic application aimed at creating isometric aerobic exercise for individuals unable to exercise voluntarily, it is desirable that the output force be minimised since otherwise measures to suppress limb movement may be required. For this reason it is proposed that the most appropriate stimulation frequency is that which maximises oxygen uptake while keeping force output to manageable levels. There are also differences in tolerability of the stimulation at different frequencies. During pilot work for the development of the protocol for this study, investigations were carried out on six subjects using continuous, as opposed to intermittent, stimulation patterns. Three of these subjects, who could readily tolerate 4 minute bouts of continuous stimulation at frequencies up to 4 Hz, could not tolerate the same amplitude of continuous stimulation at 6 Hz. The discomfort at the higher frequency increased over several minutes of the stimulation bout, and was quickly relieved upon ceasing stimulation, suggesting that it may have been related to the accumulation of metabolic by-products. The marked increase in frequency dependent discomfort appeared to coincide with the onset of partial twitch fusion.

The relative contribution of each term in Equation 7 to the overall energy rate, depicted in Figure 5, suggests that the *dTTI* component dominates in the mid frequency range, falling away at the upper end where the combination of the *f* and *TTI* terms make up most of the energy. Umberger [26] has reported the combined activation and maintenance heat rate for maximally activated isometric muscle according to the formula ${\dot{h}}_{\mathrm{am}}=1.28\;\mathrm{FT}+25$ W kg^-1^, where FT is the percentage of fast twitch fiber in the muscle. Assuming a value of FT= 65% for the quadriceps [26] and a muscle mass of 2.5 kg per leg, this would amount to some 7.7 kcal min^-1^. The total contribution of the *f* and *TTI* terms at 12 Hz in the present study was approximately a third of this value (Figure 5) and the difference may be explained in part by the 50% duty cycle which was used and the likelihood of less than maximal activation.

 The behaviour of the muscle during low frequency electrical stimulation resembles shivering and it may be the case that the energetic response is similar to thermogenic shivering, which is essentially an isometric activity for producing heat. Haman *et al.* measured the oxygen uptake with shivering during cold-exposure and demonstrated that it amounts to approximately 30% of $\dot{V}O_{2\mathrm{MAX}}$ during moderate intensity shivering [40]. They also observed that a greater proportion of carbohydrate was used than would be expected for the same intensity of ordinary exercise. The energy rate of Equation 6 is normalized to muscle mass, as distinct from the total energy estimated here by experiment. Therefore, the fitted coefficients include the unknown muscle mass as a factor. The inter-subject variance in muscle mass could therefore account for some of the energy variability not explained by the model. The intercept value may be understood to be the $\dot{E}$ with no stimulation and zero muscle output torque and therefore would be expected to be zero. The model estimates a nonzero value, however, the confidence interval includes zero which is acceptable.

The present study used bouts of stimulation lasting only 4 minutes which appeared to be sufficient to establish a steady state oxygen uptake. A useful therapeutic exercise session would need to be sustained for longer, ideally for 30 minutes or more. The interval between stimulation bouts was 4 minutes which may not have been sufficient to exclude a fatigue effect between bouts. The fatigue rate during the bouts appears to be low for stimulation frequencies up to 8 Hz, see the sample torque waveform in Figure 1a, so the recovery interval would appear to be adequate. The stimulation level used here was determined by the tolerance limit of each subject at one frequency, 12 Hz. This is unlikely to have represented the same degree of muscle activation in each subject and furthermore no information was gained about the oxygen uptake at other levels of stimulation intensity.

## Conclusions

A modified form of the classical muscle energy model can be used to account for oxygen uptake during low frequency isometric electrical stimulation and the component of the energy cost associated with force development dominates in the mid frequency range, Figure 5b. The oxygen uptake appears to be proportional to frequency up to the point at which partial twitch fusion begins; thereafter it increases at a lesser rate as twitch fusion becomes more established. The twitch fusion characteristics appear to be an important factor in selecting the optimal frequency to achieve a therapeutically significant and sustained oxygen uptake. While oxygen uptake reached a plateau at around 10 Hz, practical limitations related to user tolerance and the minimization of evoked joint torque suggest that the best frequency for producing therapeutically significant and sustainable levels of whole body oxygen utilisation is 4 to 5 Hz, which in this study was the frequency above which twitch force summation began.

## Abbreviations

ATP: Adenosine triphosphate; CE: Contractile element; COPD: Chronic obstructive pulmonary disease; dTTI: Incremental torque integral; FTI: Force time integral; MET: Metabolic equivalent; MVC: Maximum voluntary contraction; NMES: Neuromuscular electrical stimulation; NMR: Nuclear magnetic resonance; RER: Respiratory exchange ratio; Rf: Breathing frequency; SEE: Series elastic element; TTI: Torque time integral; $\dot{V}O_{2}$: Pulmonary oxygen uptake rate; $\dot{V}O_{2\mathrm{MAX}}$: Maximum pulmonary oxygen uptake rate.

## Competing interest

Author CM is an employee of Biomedical Research Ltd., who manufactured the stimulation device used in this study.

## Authors' contributions

CM conceived the research objective, devised the experimental protocol, collected and analysed the data, wrote the paper. BC reviewed the experimental protocol, reviewed the paper. ML guided and supervised the research, reviewed the paper. All authors read and approved the final manuscript.
